# Novel H5 Clade 2.3.4.4 Reassortant (H5N1) Virus from a Green-Winged Teal in Washington, USA

**DOI:** 10.1128/genomeA.00195-15

**Published:** 2015-04-02

**Authors:** Mia Kim Torchetti, Mary Lea Killian, Robert J. Dusek, Janice C. Pedersen, Nichole Hines, Barbara Bodenstein, C. LeAnn White, Hon S. Ip

**Affiliations:** a National Veterinary Services Laboratories, Veterinary Services, U.S. Department of Agriculture, Ames, Iowa, USA; b National Wildlife Health Center, United States Geological Survey, U.S. Department Of The Interior, Madison, Wisconsin, USA

## Abstract

Eurasian (EA)-origin H5N8 clade 2.3.4.4 avian influenza viruses were first detected in North America during December 2014. Subsequent reassortment with North American (AM) low-pathogenic wild-bird-origin avian influenza has generated at least two reassortants, including an EA/AM H5N1 from an apparently healthy wild green-winged teal, suggesting continued ongoing reassortment.

## GENOME ANNOUNCEMENT

H5N1 highly pathogenic avian influenza virus (HPAIV) emerged in China during 1996 and has subsequently evolved into diverse clades and subclades (1). Beginning in January 2014, a distinct group of HPAI H5 reassortant viruses (H5N8 subclade 2.3.4.4) has caused outbreaks in poultry in South Korea, and by late 2014, it had spread to Japan, the Russian Federation, and Europe, with multiple isolations occurring from wild birds, including apparently healthy birds (2). On 4 December 2014, a novel HPAI H5N2 reassortant was isolated from two poultry outbreaks in British Columbia (BC), Canada. The virus was a reassortant containing five Eurasian-origin (EA) H5N8 and three North American (AM) wild-bird-origin avian influenza virus (AIV) RNA segments (3).

A wholly Eurasian 2.3.4.4 H5N8 virus was isolated on 6 December 2014 in Whatcom County, WA, from a gyrfalcon (Falco rusticolus) that hunted and fed on a wild birds and died 2 days later (4). In the same area, a northern pintail (Anas acuta) was found dead on 8 December 2014, from which the Canadian EA/AM H5N2 reassortant virus was isolated (4). Oropharyngeal and cloacal swabs were subsequently collected from wild waterfowl as part of a hunter-harvest surveillance program to monitor the extent of H5 HPAIV transmission along the Pacific Flyway. We now report that a second reassortant virus was isolated from a green-winged teal (Anas crecca) shot by a hunter on 29 December 2014 in the same county (A/American green-winged teal/Washington/195750/2014 (H5N1)).

This novel EA/AM H5N1 reassortant virus contains 4 EA H5N8 and 4 AM-origin RNA segments. The Eurasian polymerase basic 2 (PB2), hemagglutinin (HA), nucleoprotein (NP), and matrix (MA) genes have the closest similarities (99%) to those of the A/gyrfalcon/Washington/41088-6/2014 (H5N8) and A/crane/Kagoshima/KU1/2014 (H5N8) viruses from Japan. The hemagglutinin protein has a multibasic protease cleavage site sequence of PLRERRRKR/GLF that is characteristic of the HPAIV H5 clade 2.3.4 (5). The 4 AM low-pathogenic wild-bird-lineage segments have 99% similarities to the AM AIV segments of wild-bird origin, as follows: PB1, A/bufflehead/California/3118/2011 (H4N8); polymerase acidic (PA), A/American green-winged teal/Wisconsin/11OS3425/2011 (H12N5); neuraminidase (NA), A/blue-winged teal/Texas/AI12-909/2012 (H7N1); and nonstructural (NS), A/northern shoveler/California/HKWF392sm/2007 (H10N7).

Earlier in 2014, the EA-H5N8 HPAIV circulating in South Korea was found to have diverged into two groups (A and B) (6). The group A viruses have now been detected across multiple countries, with evidence of regional diversification (intercontinental group A, subgroups 1 to 3 [*ic*A1-3; D. Lee, M. Torchetti, K. Winkler, H. Ip, C. Song, D. Swayne, unpublished data]). Two *ic*A2 reassortant viruses (EA/AM H5N2 and EA/AM H5N1) have now been detected in Washington; however, no reassortants have been detected to date in any of the other *ic*A subgroups.

The introduction of the *ic*A2-H5 clade 2.3.4.4 virus initially into the Pacific Flyway in 2014 with subsequent detection of at least two reassortants with North American low-pathogenic AIV by early 2015 suggests the potential for further reassortment events as the *ic*A2-H5 viruses continue to circulate among wild birds. Further work to monitor for such reassortments and an evaluation of these viruses are warranted. During the preparation of this paper, a nearly identical H5N1 virus was found in a backyard flock in Chilliwack, BC, Canada.

### Nucleotide sequence accession numbers.

The genome sequence of A/American green-winged teal/Washington/195750/2014 (H5N1) virus is deposited in GenBank under accession numbers KP739418 to KP739425.

## References

[1] WHO/OIE/FAO H5 Evolution Working Group 2015 Evolution of the influenza A(H5) haemagglutinin: WHO/OIE/FAO H5 Working Group reports a new clade designated 2.3.4.4. World Health Organization, Geneva, Switzerland http://www.who.int/influenza/gisrs_laboratory/h5_nomenclature_clade2344/en/.

[2] World Organisation for Animal Health (OIE) 2014 Summary of immediate notifications and follow-ups—2014 Highly path. avian influenza. World Organisation for Animal Health (OIE), Paris, France.

[3] Canadian Food Inspection Agency. 2014 Avian influenza – British Columbia infected premises. Canadian Food Inspection Agency, Ottawa, Ontario, Canada http://www.inspection.gc.ca/animals/terrestrial-animals/diseases/reportable/ai/2014-ai-investigation-in-bc/infected-premises/eng/1418340527324/1418340584180.

[4] Ip HS, Torchetti MK, Crespo R, Kohrs P, DeBruyn P, Mansfield KG, Baszler T, Badcoe L, Bodenstein B, Shearn-Bochsler V, Killian ML, Pedersen JC, Hines N, Gidlewski T, DeLiberto T, Sleeman JM 2015 Novel Eurasian highly pathogenic H5 viruses detected in wild birds from Washington state. Emerg Infect Dis. doi:10.3201/eid2105.142020. PMC4412248 25898265

[5] OFFLU OIE/FAO Network 2014 Influenza A cleavage sites. http://www.offlu.net/index.php?id=306.

[6] Jeong J, Kang HM, Lee EK, Song BM, Kwon YK, Kim HR, Choi KS, Kim JY, Lee HJ, Moon OK, Jeong W, Choi J, Baek JH, Joo YS, Park YH, Lee HS, Lee YJ 2014 Highly pathogenic avian influenza virus (H5N8) in domestic poultry and its relationship with migratory birds in South Korea during 2014. Vet Microbiol. 173:249–257. doi:10.1016/j.vetmic.2014.08.002. 25192767

